# A new minimally-invasive method for microinjection into the mouse spinal dorsal horn

**DOI:** 10.1038/srep14306

**Published:** 2015-09-21

**Authors:** Yuta Kohro, Emi Sakaguchi, Ryoichi Tashima, Hidetoshi Tozaki-Saitoh, Hideyuki Okano, Kazuhide Inoue, Makoto Tsuda

**Affiliations:** 1 Department of Molecular and System Pharmacology, Graduate School of Pharmaceutical Sciences, Kyushu University, 3-1-1 Maidashi, Higashi-ku, Fukuoka 812-8582, Japan; 2 Department of Life Innovation, Graduate School of Pharmaceutical Sciences, Kyushu University, 3-1-1 Maidashi, Higashi-ku, Fukuoka 812-8582, Japan; 3 Department of Physiology, Keio University School of Medicine, Shinjuku-ku, Tokyo 160-8582, Japan

## Abstract

Noninvasive gene delivery to the spinal dorsal horn (SDH) remains challenging because existing methods to directly microinject vectors require laminectomy, which leads to tissue damage and inflammation. Such responses might hamper accurate readouts of cellular and behavioural effects of an introduced gene. Here we develop a new minimally-invasive SDH microinjection technique without the need of laminectomy in which a microcapillary is inserted into the SDH parenchyma through an intervertebral space. Using this method, we microinjected adeno-associated virus with an astrocytic promoter into the SDH and achieved efficient gene expression in an astrocyte-specific manner without gliosis, neuronal loss or inflammation. Furthermore, astrocytic loss- and gain-of-function of the transcription factor STAT3 by expressing a dominant-negative form and a constitutive-active form of STAT3, respectively, demonstrated the necessity and sufficiency of astrocytic STAT3 in the maintenance of neuropathic pain following peripheral nerve injury, a debilitating chronic pain state in which currently available treatments are frequently ineffective. Thus, our technique enables manipulation of gene expression in cell type- and spatial-specific manners without adverse effects, and may be useful for research in SDH physiology and pathology.

The spinal dorsal horn (SDH) receives sensory information from primary afferent sensory fibres following nociceptive stimuli^1^,^2^,^3^,^4^. Information is processed in the SDH through a complex network of interneurons and projection neurons and conveyed to several regions in the brain. Furthermore, the neuronal networks in the SDH are modulated and modified under pathological conditions such as peripheral tissue inflammation and peripheral nerve injury (PNI)^1^,^4^. Recent evidence indicates that glial cells in the SDH contribute to these pathologically altered neurotransmissions^5^,^6^. Thus, elucidating the roles of each cell type and understanding the communication between neurons and glial cells in the SDH will advance our understanding of the mechanisms involved in the activation, modulation and modification of sensory processing in the SDH under physiological and pathological conditions. Several approaches have been established to regulate gene expression, such as intrathecal injection of antisense oligonucleotides or siRNA and the conditional deletion of a gene using the Cre-loxP system. However, technical limitations of these methods include lack of segment-, region- and cell type-specificity, or the conditional deletion of genes at the developmental stage. Recently, several viral vectors have been reported to be useful tools for region- and cell type-specific gene transduction^7^,^8^,^9^. Several groups have tried to introduce genes into target cells in mice and rats using an intra-spinal injection method^10^,^11^,^12^,^13^,^14^,^15^,^16^. However, existing methods need complicated surgical procedures such as laminectomy^10^,^11^,^12^,^13^,^14^ or drilling of a hole in the vertebra^15^,^16^ for direct access to the spinal cord parenchyma, which results in an inflammatory response and extensive tissue damage that could hamper cellular and behavioural phenotypes of sensory processing. Indeed, recent studies have shown that inflammatory factors such as pro-inflammatory cytokines affect neuronal excitability and pain behaviours^17^,^18^,^19^.

To overcome the limitations described above, we set out to establish a new microinjection technique into the SDH. In this study, we developed a minimally-invasive SDH microinjection technique in mice without the need of laminectomy and any complicated surgical procedures: a microcapillary is inserted into the SDH parenchyma through an intervertebral space. This method is much easier and less invasive than previously reported methods. Furthermore, using this new technique, we successfully introduced genes using recombinant adeno-associated viral (rAAV) vectors into 4th lumbar (L4)-SDH astrocytes without any apparent detrimental effects. In addition, we provide the first evidence indicating the necessity and sufficiency of astrocytic signal transducer and activator of transcription 3 (STAT3) in the maintenance of neuropathic pain. Thus, our established microinjection technique enables the manipulation of gene expression in the SDH in a cell type- and segment-specific manner, without adverse effects, and may be useful for basic research in SDH physiology and pathology.

## Results

### Establishment of a minimally invasive intra-SDH injection method

**Figure 1:**
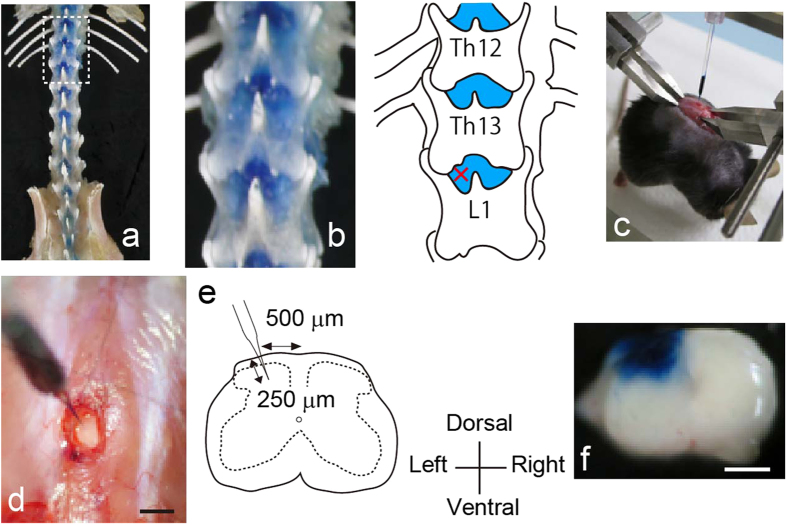
New minimally invasive method for intra-spinal dorsal horn (SDH) microinjection. (**a**) Skeletal preparation of mouse vertebral column and pelvis viewed from the dorsal aspect. (**b**) High magnification of dashed line in the panel (**a**) and its schematic illustration. Blue stained regions are uncovered by vertebra. X; intra-SDH microinjection site. (**c**) Photograph showing intra-SDH microinjection. The mouse was fixed by attaching custom-made clamps to the vertebral column. A microcapillary was filled with Evans blue. (**d**) High magnification view of intra-SDH injection site (Scale bar, 1 mm). (**e**) Schematic illustration of intra-SDH injection. (**f**) Spinal cord section of mouse after intra-SDH microinjection of Evans blue (Scale bar, 500 μm).

To search for a site enabling intra-SDH microinjection without laminectomy, we first visualized the skeleton of the vertebrae at the thoracic and lumbar levels of the mouse that had been fixed and injected intrathecally with Evans blue (Fig. 1a). We found that there was a dense blue area at each interspace between vertebrae that was not covered by the spine (Fig. 1b). Next, we determined if microinjection through this intervertebral space enabled delivery of substances into the SDH without laminectomy. An incision was made in the back of an anaesthetized mouse placed in a stereotaxic apparatus (Fig. 1c) and a small opening in the muscles around the left side of the interspace between Th13 and L1 vertebrae (Fig. 1d) was made. The dura mater and the arachnoid membrane were incised to make a small window to allow insertion of the microcapillary directly into the SDH. We inserted the microcapillary into the SDH (250 μm in depth from the surface of the dorsal root entry zone; Fig. 1d,e) and injected the dye. After injection, we removed the lumbar spinal cord and confirmed that the injected dye was successfully localized in the SDH (Fig. 1f). Thus, this technique enabled injection of a substance into the SDH without laminectomy in the mouse.

### Specific transgene expression in SDH astrocytes by intra-SDH injection of rAAV

**Figure 2:**
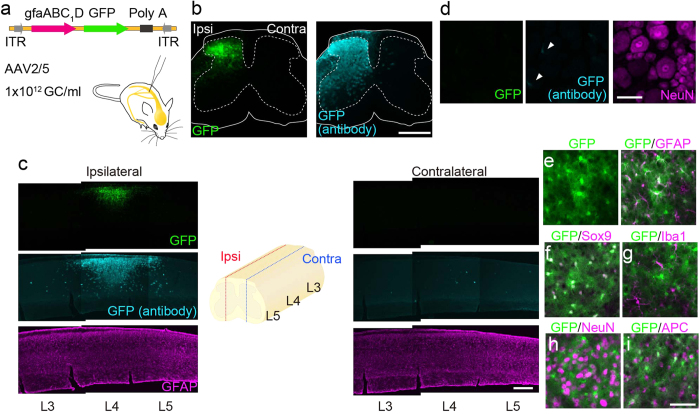
Astrocyte-specific gene expression by intra-SDH microinjection of AAV2/5-gfaABC_1_D. (**a**) Schematic illustration of a viral vector construct. (**b**,**c**) GFP expression in the spinal cord of mice 28 days after intra-SDH injection of AAV2/5-gfaABC_1_D-GFP (**b** transverse; **c** sagittal sections). GFAP (magenta) was used to counterstain the spinal cord. Ipsi, ipsilateral; C contralateral (Scale bar, 500 μm). (**d**) GFP expression in the 4th lumbar (L4) dorsal root ganglion (DRG) of intra-SDH injected mice. NeuN (magenta) was used to counterstain the DRG (Scale bar, 50 μm). (**e–i**) Immunohistochemical identification of GFP-expressing cells using cell-type markers (magenta; **e** GFAP; **f** Sox9; **g** Iba1; **h** NeuN; **i** APC) in the L4-SDH sections 28 days after intra-SDH injection of AAV2/5-gfaABC_1_D-GFP (Scale bar, 50 μm).

Using this new technique, we attempted to express genes in the SDH in a cell type-specific manner. In this study, we focused on astrocytes, the most abundant cell-type in the central nervous system (CNS), because they play a pivotal role in neuronal function under physiological and pathological conditions^20^,^21^,^22^, including chronic pain. We microinjected AAV2/5 (a serotype showing a preferential tropism for astrocytes^23^) containing a gene encoding green fluorescent protein (GFP) under the control of a *gfap* promoter (gfaABC_1_D; which increases astrocyte-specific expression^24^,^25^,^26^,^27^) (AAV2/5-gfaABC_1_D-GFP; Fig. 2a) unilaterally into the SDH through the window on the interspace between Th13 and L1 vertebrae. Twenty-eight days later, we observed strong GFP fluorescence exclusively in the ipsilateral SDH, but not contralateral to the injection site (Fig. 2b). GFP expression was predominantly localized in the L4 segment of the SDH (Fig. 2c). Consistent with these results, a similar spatial localization of GFP immunofluorescence was detected with an antibody directed against GFP (Fig. 2b,c). In contrast, we did not observe GFP fluorescence in dorsal root ganglion (DRG) neurons and only observed faint GFP immunofluorescence with the antibody (Fig. 2d, arrowheads). To determine the cell type-specificity of transgene expression, we performed immunolabelling using cell type-specific markers for identification of GFP-expressing cells. Almost all GFP-positive cells double-labelled with glial fibrillary acidic protein (GFAP) (Fig. 2e) and Sox9 (Fig. 2f), both of which are markers of astrocytes: 99.0% of total GFP-positive cells had Sox9 immunofluorescence, and 73.5% of Sox9-positive cells were positive to GFP (n = 5), indicating that the majority of total SDH astrocytes were efficiently transduced. We also observed no co-localization of GFP with ionized calcium-binding adapter molecule-1 (Iba1)- (Fig. 2g), neuronal nuclei (NeuN)- (Fig. 2h) or adenomatous polyposis coli (APC)-positive cells (Fig. 2i). Therefore, these results indicate that intra-SDH microinjection of AAV2/5-gfaABC_1_D-GFP induces selective gene expression in L4-SDH astrocytes.

### Titre- and time-dependent inflammation and alteration in pain responses by intra-SDH injection of AAV2/5

**Figure 3:**
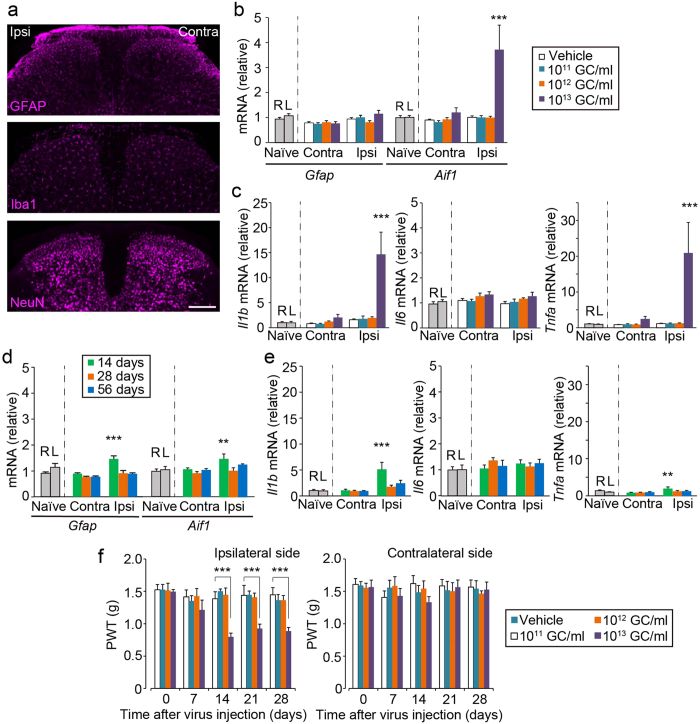
Titre-dependent adverse effects following intra-SDH AAV2/5-gfaABC_1_D injection. (**a**) Immunofluorescence labelling for cell-type markers (GFAP, astrocytes; Iba1, microglia; NeuN, neurons) in the L4-SDH sections 28 days after intra-SDH injection of AAV2/5-gfaABC_1_D-GFP (Scale bar, 200 μm). (**b**,**c**) mRNA expression of genes 28 days after intra-SDH injection of vehicle or rAAV (10^11^–10^13^ GC/ml), and in naïve mice. Values represent the relative ratio of mRNA (normalized to *Gapdh* mRNA) to the mean expression level of mRNA in naïve mice. R, right; L, left (**b**,**c**; n = 6–8, ^***^P < 0.001 versus naïve spinal cord). (**d**,**e**) mRNA expression of genes 14, 28 and 56 days after intra-SDH injection of rAAV (10^12^ GC/ml) and in naïve mice. Values represent the relative ratio of mRNA (normalized to *Gapdh* mRNA) to the mean expression level of mRNA in naïve mice. (**d**,**e**; n = 6–8, ^**^P < 0.01; ^***^P < 0.001 versus naïve spinal cord). (**f**) Paw withdrawal threshold (PWT) of mechanical stimulation to the ipsilateral and contralateral hindpaws of vehicle and virus injected mice (10^11^–10^13^ GC/ml) before (day 0) and after intra-SDH injection (n = 8; ^***^P < 0.001 versus vehicle injected group).

A previous study has shown that AAV2/5-transduced astrocytes become activated in a titre-dependent manner^23^. However, under our conditions in which AAV2/5-gfaABC_1_D-GFP [10^12^ genome copies (GC)/ml] was injected, neither gliosis (astrocytes and microglia) nor neuronal loss in the L4-SDH were observed (Fig. 3a). Consistent with this result, there was no change in the expression GFAP (*Gfap*) and Iba1 (*Aif1*) mRNA in the SDH (Fig. 3b). Furthermore, the levels of mRNA for interleukin (IL)-1β (*Il1b*), IL-6 (*Il6*) and tumor necrosis factor (TNF)-α (*Tnfa*) were not elevated (Fig. 3c). In contrast, a higher titre (10^13^ GC/ml) of AAV2/5-gfaABC_1_D-GFP not only increased the expression of GFP (Supplementary Fig. 1a), but also *Aif1*, *Il1b* and *Tnfa*, suggesting that this titre induces an inflammatory response (Fig. 3b,c). Furthermore, we investigated the time-dependent inflammatory response after SDH microinjection of 10^12^ GC/ml of AAV2/5-gfaABC_1_D-GFP. While a slight upregulation of *Gfap*, *Aif1*, *Il1b* and *Tnfa* mRNA was observed 14 days after intra-SDH injection, their expression returned to basal levels over the next 14 or 42 days (Fig. 3d,e). GFP expression was not different on either day 14 or day 28 after injection and significantly increased on day 56 (Supplementary Fig. 1b).

To examine the influence of these viral infection and inflammatory events on animal behaviour *in vivo*, we measured paw withdrawal threshold (PWT) to mechanical stimulation, which was applied to the hindpaw. Mice injected with AAV2/5-gfaABC_1_D-GFP (10^11^ or 10^12^ GC/ml) into the SDH showed no significant change in PWT (Fig. 3f). In contrast, the high titre of AAV2/5-gfaABC_1_D-GFP (10^13^ GC/ml) resulted in a marked decrease in PWT of the ipsilateral side from 14 days after intra-SDH injection, but not on the contralateral side (Fig. 3f). Therefore, under our experimental conditions, 28 days after SDH microinjection of 10^12^ GC/ml viral-titre of AAV2/5-gfaABC_1_D-GFP was an optimal condition to introduce a gene of interest into SDH astrocytes without an inflammatory response or sensory abnormalities.

### Astrocytic STAT3 in the SDH is required for maintaining neuropathic pain

**Figure 4:**
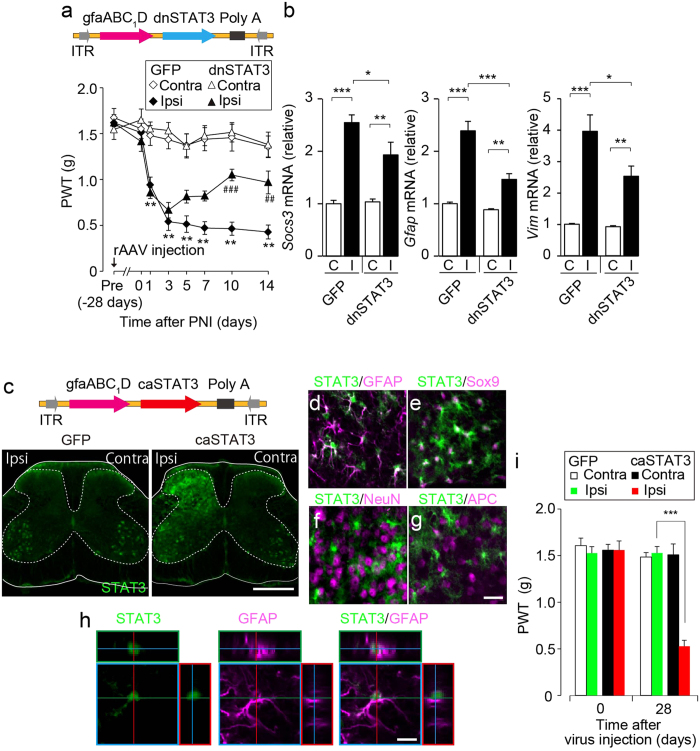
The L4-SDH astrocytic STAT3 signalling is necessary and sufficient for the maintenance of neuropathic pain. (**a**) PWT of C57BL/6J mice injected with AAV2/5-gfaABC_1_D-GFP or -dnSTAT3 pre (−28 days), before (day 0) and after peripheral nerve injury (PNI) (n = 8, ^**^P < 0.01 versus pre, ^##^*P* < 0.01; ^###^*P* < 0.001 versus ipsilateral side of AAV2/5-gfaABC_1_D-GFP-injected group). (**b**) Real-time PCR analysis of mRNA in mice injected with AAV2/5-gfaABC_1_D-GFP or -dnSTAT3 14 days after PNI. Values represent the relative ratio of mRNA (normalized to *actb* mRNA) to the contralateral side of AAV2/5-gfaABC_1_D-GFP injected group. C, contralateral; I, ipsilateral. (n = 7–8, ^*^P < 0.05; ^**^P < 0.01; ^***^P < 0.001). (**c**) Immunofluorescence of STAT3 in L4 spinal cord sections 28 days after intra-SDH injection of AAV2/5-gfaABC_1_D-GFP or -caSTAT3 (Scale bar, 500 μm). (**d-g**) Double immunofluorescence labelling for STAT3 (green) and cell-type markers (magenta; **d** GFAP; **e** Sox9; **f** NeuN; **g** APC) in L4-SDH sections 28 days after intra-SDH injection of AAV2/5-gfaABC_1_D-GFP or -caSTAT3 (Scale bar, 20 μm). (**h**) Representative confocal z-stack digital images of a single cell double-immunolabelled with STAT3 (green) and GFAP (magenta). Scale bar, 10 μm. (**i**) PWT of AAV2/5-gfaABC_1_D-GFP- or -caSTAT3-injected mice before (day 0) and after virus injection (n = 8, ^***^P < 0.001 versus ipsilateral side of AAV2/5-gfaABC_1_D-GFP injected group).

We have previously shown that PNI causes activation of STAT3 in spinal astrocytes and that intrathecal administration of an inhibitor of JAK alleviates the developed pain hypersensitivity after PNI^28^. These results suggest the involvement of the astrocytic JAK-STAT3 signalling pathway in the maintenance of neuropathic pain. In contrast, other studies have reported that STAT3 signalling in spinal microglia contributes to neuropathic pain development^16^,^29^. Therefore, the role of astrocytic STAT3 in neuropathic pain remains controversial. To clarify this, we utilized the established intra-SDH microinjection technique to express a dominant-negative form of STAT3 (dnSTAT3)^30^ in a SDH astrocyte-specific manner (Fig. 4a).

AAV2/5-gfaABC_1_D-dnSTAT3-treated mice showed no effect of basal mechanical sensitivity before PNI (Fig. 4a). Following PNI, PWT decreased, which was comparable between mice injected with AAV2/5-gfaABC_1_D-GFP and -dnSTAT3 from day 1 to day 3 (Fig. 4a). However, AAV2/5-gfaABC_1_D-dnSTAT3-treated mice produced a progressive recovery in decreased PWT from day 5 to day 14 (Fig. 4a). Furthermore, PNI-induced upregulation of suppressor of cytokine signalling 3 (*Socs3*), *Gfap* and vimentin (*Vim*) on day 14 was significantly suppressed by introducing dnSTAT3 in L4-SDH astrocytes (Fig. 4b). These results indicate that interfering with astrocytic STAT3 activation suppresses the maintenance of PNI-induced tactile allodynia. To validate the effect of astrocytic introduction of dnSTAT3, we used a conditional knockout approach in which we crossed *Stat3*^*fl/fl*^ mice with transgenic mice bearing Cre recombinase under the control of the GFAP promoter (*Gfap-cre* mice) and generated *Gfap-cre*;*Stat3*^*fl/fl*^ mice in which the loss of STAT3 function occurs preferentially in astrocytes^31^,^32^. Consistent with the result of AAV2/5-gfaABC_1_D-dnSTAT3-injected mice, *Gfap-cre*;*Stat3*^*fl/fl*^ mice displayed significant recovery of PNI-induced tactile allodynia (Supplementary Fig. 2a) and a marked suppression of PNI-induced upregulation of *Socs3*, *Gfap* and *Vim* (Supplementary Fig. 2b). Together, these results strongly suggest that L4-SDH astrocytic STAT3 signalling is necessary for the transition of astrocytes to a reactive state and for the maintenance of neuropathic pain after PNI.

We then examined whether STAT3 activation in SDH astrocytes produces tactile allodynia in mice without nerve injury. To induce gain of function of STAT3 in SDH astrocytes, we microinjected AAV2/5 with the constitutive active form of STAT3 (caSTAT3)^33^ into the SDH (Fig. 4c). Strong STAT3 immunofluorescence was observed in the L4-SDH 28 days after intra-SDH injection of AAV2/5-gfaABC_1_D-caSTAT3 compared with AAV2/5-gfaABC_1_D-GFP (Fig. 4c). Double-immunolabelling experiments using cell markers revealed that caSTAT3-positive cells were double-labelled with GFAP (Fig. 4d,h) and Sox9 (Fig. 4e), but not with NeuN (Fig. 4f) and APC (Fig. 4g). Under such conditions, mice injected with AAV2/5-gfaABC_1_D-caSTAT3 showed a significant decrease in PWT on the ipsilateral side (Fig. 4i). These results imply that L4-SDH astrocytic STAT3 activation is sufficient to produce tactile allodynia.

## Discussion

In the current study, we established a new technique for intra-SDH microinjection in mice. This method does not need any complicated surgical procedures such as laminectomy or drilling of a hole in the vertebra, which are commonly necessary for intra-spinal injection. In our method, both surgical operation and microinjection into the SDH was completed on average within 15 min per mouse. Thus, our established method is much easier and faster than that of existing methods and enables microinjection of substances into the mouse SDH without any substantial tissue and SDH damage. This minimally invasive method enables microinjection of a variety of substances into the SDH. Here, we showed cell type- and segment-specific expression of genes of interest in the SDH. In this study, by using the astrocyte tropic AAV serotype AAV2/5 with the *gfap* promoter gfaABC_1_D, we achieved efficient and selective gene transduction in L4-SDH astrocytes. In addition, intra-SDH microinjection did not lead to gene expression in the DRG. Using a viral titre of 10^12^ GC/ml, almost all transduced cells expressed the astrocytic marker Sox9, and over 70% of total astrocytes in the L4-SDH were transduced. The transduction efficiency (and also gene expression per cell) could be increased by intra-SDH injection of AAV2/5 at higher viral titres. Indeed, a high titre (10^13^ GC/ml: approximately 5–6 × 10^9^ GC per injection) of AAV2/5-gfaABC_1_D resulted in increased GFP expression. However, it should be noted that microinjection of this viral titre led to a marked increase in the expression of the microglial marker Iba1 and proinflammatory cytokines in the SDH, implying that inflammatory responses might be induced. The detrimental responses also affected animal behaviour, because mice displayed long-lasting pain hypersensitivity. Therefore, the viral titre is a critical factor when determining the effect of an introduced gene on behavioural phenotypes. However, it appears that titre-dependent adverse effects of intraparenchymal injection of rAAV may vary depending on the method, site of injection, or vector construction. Intra-hippocampal injection of AAV2/5-gfa104-eGFP (3 × 10^10^ GC per injection) induces reactive astrocytosis without microgliosis^23^. Injection of AAV2/5-gfaABC_1_D-cyto-GCaMP3 (3 × 10^10^ GC per injection) and Lck-GCaMP3 (2.4 × 10^10^ GC per injection) into the hippocampus did not cause astrogliosis^24^. However, these studies have not measured the expression of proinflammatory factors after viral infection. In contrast, 10^12^ GC/ml of AAV2/5-gfaABC_1_D-GFP, which resulted in efficient and selective gene expression in L4-SDH astrocytes, did not affect behavioural sensitivity to mechanical stimulation over the 1 month of testing after the injection, however, a slight increase in the expression of proinflammatory cytokines, *Aif1* and *Gfap* were observed on day 14. Thus, we conclude that intra-SDH microinjection of AAV2/5-gfaABC_1_D at 10^12^ GC/ml of viral titre would be optimal to enable efficient and selective gene transduction in L4-SDH astrocytes without detrimental inflammatory responses or sensory abnormalities.

Spinal astrocytes have been proposed to play an important role in the maintenance of neuropathic pain based on studies where manipulation of the function or expression of molecules activated in spinal astrocytes (such as the transcription factor STAT3) by intrathecal administration of either pharmacological inhibitors, antisense oligodeoxynucleotides or siRNAs^28^,^34^,^35^,^36^,^37^,^38^,^39^ affects established neuropathic pain after PNI. However, there is no direct evidence showing that SDH astrocyte-specific gene manipulation modulates neuropathic pain maintenance. In addition, the involvement of STAT3 in spinal astrocytes has remained controversial because it has been reported that PNI activates STAT3 in astrocytes and microglia and that intrathecal administration of an inhibitor of JAK, which is an upstream activator of STAT3, suppresses the induction or established tactile allodynia after PNI in rats^16^,^28^,^29^. In this study, by using our established astrocyte-specific gene expression method, we demonstrated for the first time the pivotal role of astrocytic STAT3 in the SDH for the maintenance of neuropathic pain. We showed that expression of dnSTAT3 in L4-SDH astrocytes produced a progressive reversal of established tactile allodynia after PNI. This reversal is supported by our previous study showing that pharmacological inhibition of JAK reduces established allodynia after PNI in rats^28^. Interestingly, despite the expression of dnSTAT3 in L4-SDH astrocytes prior to PNI, the induction of tactile allodynia was not affected. This finding strengthens the role of astrocytes in the maintenance phase. Furthermore, the reversal of PNI-induced tactile allodynia and astrocytic gene expression in mice with L4-SDH astrocyte-specific expression of dnSTAT3 was also observed in *Gfap-cre*;*Stat3*^*fl/fl*^ mice. Cre recombinase activity in *Gfap-cre*;*Stat3*^*fl/fl*^ mice occurs not only in SDH astrocytes but also other regions including ventral spinal cord astrocytes and DRG satellite glia (Supplementary Fig. 3). Therefore, this similar phenotype obtained from two independent approaches provides compelling evidence indicating that astrocytic STAT3 in the L4-SDH is necessary for maintaining tactile allodynia. In addition, it is conceivable that the observed phenotype in *Gfap-cre*;*Stat3*^*fl/fl*^ mice may be associated with STAT3 inactivation in L4-SDH astrocytes. This view is supported by our gain-of-function approach in which L4-SDH-specific introduction of caSTAT3 alone produced tactile allodynia in normal mice. Collectively, our findings from astrocyte-specific loss- and gain-of-function approaches and conditional knockout mice strongly indicate that STAT3 in L4-SDH astrocytes is not only necessary for maintaining tactile allodynia but is also sufficient to induce allodynia.

Our established intra-SDH microinjection technique is useful to investigate the role of a gene of as-yet-unknown function or of disease-related mutation reported in humans in neuropathic pain and other sensory processing. This technique also yields a versatile method adaptable for multiple applications. Applying this method for optogenetics^40^,^41^ or chemogenetics (designer receptors exclusively activated by designer drugs; DREADD^42^) approaches by expressing genes such as for channelrhodopsin-2 or synthetic G-protein-coupled receptors, respectively, can specifically control astrocytic activity in the L4-SDH *in vitro* and *in vivo*. This would be a powerful tool for revealing new insights into the role of astrocytes in the SDH, including their contribution to pain and other sensory processing. Furthermore, by utilizing a promoter for neurons or other glial cells, it will allow the introduction of genes of interest into other cell types in the L4-SDH. Thus, combination of our new intra-SDH microinjection technique with recent genetic technologies would be a useful method to advance our understanding of spinal cord physiology and pathology.

## Methods

### Animals

C57BL/6J Jcl mice (Clea Japan, Tokyo, Japan), *Gfap-cre* mice (line 73.12, The Jackson Laboratory, ME, USA) and *Stat3*^*fl/fl*^ mice^43^ were used. *Stat3*^*fl/fl*^ mice were crossed with *Gfap-cre* mice to generate *Gfap-cre*;*Stat3*^*fl/fl*^ mice and *Stat3*^*fl/fl*^ littermates were used as a control. To explore the specificity of Cre recombinase activity in *Gfap-cre* mice, *Gfap-cre* mice were crossed with CAG-CAT^fl/fl^-EGFP (CCE) mice, a reporter mouse line having a CAG [cytomegalovirus (CMV) early enhancer element and chicken β-actin] promotor, followed by a loxP-flanked chloramphenicol acetyltransferase (CAT) sequence upstream of EGFP. We used male C57BL/6J mice and male or female *Stat3*^*fl/fl*^, *Gfap-cre*; *Stat3*^*fl/fl*^ mice and *Gfap-cre*; *CCE* mice. All mice used were 8–12 weeks of age at the start of each experiment and were housed at 22 ± 1 °C with a 12-h light-dark cycle. All animals were fed food and water *ad libitum*. All animal experiments were conducted according to relevant national and international guidelines contained in the ‘Act on Welfare and Management of Animals’ (Ministry of Environment of Japan) and ‘Regulation of Laboratory Animals’ (Kyushu University) and under the protocols approved by the Institutional Animal Care and Use committee review panels at Kyushu University.

### Recombinant adeno-associated virus (rAAV) vector production

To produce rAAV vector for astrocyte specific gene transduction, a vector containing the astrocytic promoter gfaABC_1_D^44^ was generated from pZac2.1 by substituting the CMV promoter with gfaABC_1_D (amplified from addgene #19974). We then cloned AcGFP (referred to as GFP), the dominant negative form of STAT3 (dnSTAT3, amplified from addgene #8709) and the constitutive active form of STAT3 (caSTAT3, amplified from addgene #13373) into the above modified pZac2.1 to generate pZac2.1-gfaABC_1_D-GFP, pZac2.1-gfaABC_1_D-dnSTAT3 and pZac2.1-gfaABC_1_D-caSTAT3, respectively. The rAAV vector was produced from human embryonic kidney 293 (HEK293) cells with triple transfection [pZac, cis plasmid; pAAV2/5, trans plasmid; pAd DeltaF6, adenoviral helper plasmid (All plasmids were purchased from the University of Pennsylvania Gene Therapy Program Vector Core)]^45^,^46^ and purified by two cesium chloride density gradient purification steps^47^. The vector was dialyzed against phosphate-buffered saline (PBS) containing 0.001% (v/v) Pluronic-F68 using Amicon Ultra 100K filter units (Millipore, Darmstadt, Germany). The Genome titre of rAAV was determined by Pico Green fluorometric reagent (Molecular Probes, OR, USA) following denaturation of the AAV particle. Vectors were stored in aliquots at −80 °C until use.

### Skeletal preparations of mouse vertebral column

Under isoflurane [2% (v/v)] anaesthesia, 1% (w/v) Evans blue (10 μL) was intrathecally injected through the L5–L6 intervertebral space^48^ to visualize the skeleton of the vertebrae. After several minutes, mice were deeply anaesthetized by intraperitoneal (i.p.) injection of pentobarbital and perfused transcardially with PBS, followed by ice-cold 4% (w/v) paraformaldehyde/PBS. The vertebral column was removed and further dissection was performed to clearly visualize the vertebral, sacral and medial iliac bones by removing the muscles or other connective tissue. The dissected preparation was briefly bleached and photographed (Fig. 1a,b).

### Intra-SDH injection of dye or rAAV vector

Mice were deeply anaesthetized by subcutaneous (s.c.) injection of ketamine (100 mg kg^−1^) and xylazine (10 mg kg^−1^). The skin was incised at Th12–L3, and custom-made clamps were attached to the rostral and caudal sites of the vertebral column (Fig. 1c). Paraspinal muscles around the left side of the interspace between Th13 and L1 vertebrae were removed, and the dura mater and the arachnoid membrane were carefully incised using the tip of a 30G needle to make a small window to allow the microcapillary Femtotip (Eppendorf, NY, USA) insert directly into the SDH (Fig. 1d). The microcapillary was inserted with a pre-flow of 1% (w/v) Evans blue or rAAV solution through the small window (approximately 500 μm lateral from the midline) and inserted into the SDH (250 μm in depth from the surface of the dorsal root entry zone) (Fig. 1e). Evans blue or rAAV solution was pressure-ejected (600 hPa) for 50–60 seconds (approximately 500–600 nL) using the FemtoJet Express (Eppendorf, NY, USA). After microinjection, the inserted microcapillary was removed from the SDH, the skin was sutured with 3-0 silk, and mice were kept on a heating pad until recovery.

### Peripheral nerve injury

We used the spinal nerve injury model^49^ with some modifications^50^. Under isoflurane (2%) anaesthesia, a small left-side incision at L3-S1 was made. Paraspinal muscles and fats were removed from L5 traverse process, and the part of this traverse process was removed to expose the parallel-lying L3 and L4 spinal nerves. The L4 nerve was carefully isolated and cut. The wound was sutured with 3-0 silk. The surrounding skin was pulled together and sutured with 3-0 silk.

### Immunohistochemistry

Mice were deeply anaesthetized by i.p. injection of pentobarbital and perfused transcardially with PBS, followed by ice-cold 4% paraformaldehyde/PBS. The L4 (transverse) or L3–L5 (sagittal) segments of the spinal cord, or the L4 DRG were removed, postfixed in the same fixative for 3 h at 4 °C, and placed in 30% sucrose solution for 24 h at 4 °C. Transverse L4 spinal cord sections (30 μm), sagittal L3-L5 spinal cord sections (30 μm) and L4 DRG sections (15 μm) were incubated in blocking solution (3% normal goat serum or normal donkey serum) for 2 h at room temperature and then incubated for 48 h at 4 °C with primary antibodies: polyclonal rabbit anti-GFP (1:2000, MBL, Aichi, Japan), polyclonal rabbit anti-STAT3 (1:1000, Cell signalling, MA, USA), polyclonal goat anti-Sox9 (1:1000, R&D Systems, MN, USA), monoclonal rat anti-GFAP (1:1000, Invitrogen, CA, USA), monoclonal mouse anti-NeuN (1:200, Millipore, Darmstadt, Germany), polyclonal rabbit anti-Iba1 (1:5000, Wako, Osaka, Japan), and monoclonal mouse anti-APC (CC1 clone, 1:500, Calbiochem, CA, USA). Following incubation, tissue sections were washed and incubated for 3 h at room temperature in secondary antibody solution (Alexa Fluor 488 and/or 546, 1:1000, Molecular Probes, OR, USA and/or CF 405, 1:1000, Biotium, CA, USA). The tissue sections were washed, slide-mounted and subsequently coverslipped with Vectashield hardmount (Vector Laboratories, PA, USA). Three to five sections from the L4 spinal cord segments of each mouse were randomly selected and analysed using an LSM510 and 700 Imaging System (Carl Zeiss, Oberkochen, Germany).

### Behavioural studies

To assess tactile allodynia, mice were placed individually in an opaque plastic cylinder, which was placed on a wire mesh and habituated for 1 h to allow acclimatization to the new environment. After that, calibrated von Frey filaments (0.02–2.0 g, North Coast Medical, CA, USA) were applied to the plantar surfaces of the hindpaws of mice with or without intra-SDH injection, or PNI from below the mesh floor and the 50% PWT was determined using the up-down method^51^.

### Quantitative real-time PCR

Mice were deeply anaesthetized with pentobarbital, perfused transcardially with PBS, and L3-L4 spinal cord was removed and separated half at middle of the spinal cord by a sagittal cut and then into dorsal and ventral horn by a horizontal cut, so that a sample contained only dorsal horn of the ipsilateral or contralateral side to the virus injection or PNI. Total RNA from the L3–L4 SDH was extracted using Trisure (Bioline, London, UK) according to the manufacturer’s protocol and purified with RNeasy mini plus kit (Qiagen, CA, USA). The amount of total RNA was quantified by measuring the OD_260_ using a Nanodrop spectrophotometer (Nanodrop, DE, USA). For reverse transcription, 200 ng of total RNA was transferred to the reaction with Prime Script reverse transcriptase (Takara, Kyoto, Japan). Quantitative polymerase chain reaction (PCR) was carried out with FastStart Essential DNA Probe Master (Roche, BSL, Switz) using a LightCycler 96 or a LightCycler 480 system (Roche, BSL, Switz). Expression levels were normalized with to GAPDH (glyceraldehyde-3-phosphate dehydrogenase) or β-actin. The sequence of TaqMan probe and forward and reverse primers used in this study were described below.

GFP: 5′-FAM-ACCCCAACGAGAAGCGCGATCA-TAMRA-3′ (probe), 5′-TGTGCTGCTGCCCGATAA-3′ (forward primer),

5′-GTCACGAAGCCGAAGTAGATCA-3′ (reverse primer).

Iba1 (Aif1): 5′-FAM-CAGGAAGAGAGGCTGGAGGGGATCAA-TAMRA-3′ (probe), 5′-GATTTGCAGGGAGGAAAAGCT-3′ (forward primer),

5′-AACCCCAAGTTTCTCCAGCAT-3′ (reverse primer).

GFAP: 5′-FAM-CGTCCCGCAACGCAGAGCTG-TAMRA-3′ (probe), 5′-GAGTGGTATCGGTCTAAGTTTGCA-3′ (forward primer),

5′-GCGGCGATAGTCGTTAGCTT-3′ (reverse primer).

Vimentin (Vim): 5′-FAM-TTGACACCCACTCAAAAAGAACACTCCTGA-TAMRA-3′ (probe), 5′-CCCTGAACCTGAGAGAAACTAACC-3′ (forward primer),

5′-GTCTCATTGATCACCTGTCCATCT-3′ (reverse primer).

IL-6: 5′-FAM-TCACAGAGGATACCACTCCCAACAGACCTG-TAMRA-3′ (probe), 5′-GGGACTGATGCTGGTGACAA-3′ (forward primer),

5′-TGCCATTGCACAACTCTTTTCT-3′ (reverse primer).

TNF-α: 5′-FAM-TACGTGCTCCTCACCCACACCGTCA-TAMRA-3′ (probe), 5′-GTTCTCTTCAAGGGACAAGGCTG-3′ (forward primer),

5′-TCCTGGTATGAGATAGCAAATCGG-3′ (reverse primer).

IL-1β: 5′-FAM-TGCAGCTGGAGAGTGTGGATCCCAA-TAMRA-3′ (probe), 5′-GAAAGACGGCACACCCACC-3′ (forward primer),

5′-AGACAAACCGCTTTTCCATCTTC-3′ (reverse primer).

SOCS3: 5′-FAM-CAGCTCGGACCAGCGCCACTTCT-TAMRA-3′ (probe), 5′-CGCGGGCACCTTTCTTATC-3′ (forward primer),

5′-CTCACACTGGATGCGTAGGTTCT-3′ (reverse primer).

GAPDH: 5′-FAM-ACCACCAACTGCTTAGCCCCCCTG-TAMRA-3′ (probe), 5′-TGCCCCCATGTTTGTGATG-3′ (forward primer),

5′-GGCATGGACTGTGGTCATGA-3′ (reverse primer).

β-actin (Actb): 5′-FAM- CCTGGCCTCACTGTCCACCTTCCA-TAMRA-3′ (probe), 5′-CCTGAGCGCAAGTACTCTGTGT-3′ (forward primer),

5′-CTGCTTGCTGATCCACATCTG-3′ (reverse primer).

### Statistical analysis

All data are shown as the mean ± SEM. Statistical significance of differences was determined using two-way ANOVA with *post hoc* Bonferroni test (Figs 3f and 4a,i), one-way ANOVA with *post hoc* Dunnett’s multiple comparison test (Fig. 4a) or Tukey’s multiple comparison test (Figs 3b–e and 4b) using GraphPad Prism 4 software. Differences were considered significant at *P* < 0.05.

## Additional Information

**How to cite this article**: Kohro, Y. *et al.* A new minimally-invasive method for microinjection into the mouse spinal dorsal horn. *Sci. Rep.* **5**, 14306; doi: 10.1038/srep14306 (2015).

## Supplementary Material

Supplementary Information
