# Supplementary material for: A new minimally-invasive method for microinjection into the mouse spinal dorsal horn
Source: Sci Rep. 2015 Sep 21;5:14306. doi: 10.1038/srep14306 (PMC4585681; doi:10.1038/srep14306)
Supplement: Supplementary Information [file srep14306-s1.pdf]

Supplementary information

A new minimally-invasive method for microinjection into the mouse spinal dorsal horn

Yuta Kohro, Emi Sakaguchi, Ryoichi Tashima, Hidetoshi Tozaki-Saitoh, Hideyuki Okano, Kazuhide Inoue, Makoto Tsuda

Supplementary Figure 1 Titer- and time-dependent transgene expression by intra-SDH injection of AAV2/5-gfaABC$_1$D-GFP

(a) mRNA expression of GFP 28 days after intra-SDH injection of vehicle or rAAV (10^{11}–10^{13} GC/ml). Values represent the relative ratio of mRNA (normalized to *Gapdh* mRNA) to the ipsilateral side of 10^{11} GC/ml injected group (n=6-8, $^{\#\#}P<0.01$ versus ipsilateral side of 10^{11} GC/ml injected group). (b) mRNA expression of GFP 14, 28 and 56 days after intra-SDH injection of rAAV (10^{12} GC/ml). Values represent the relative ratio of mRNA (normalized to *Gapdh* mRNA) to the ipsilateral side of 14 days after virus-injected group (n=6-8, $^{\#\#}P<0.01$ versus ipsilateral side of 14 days after virus-injected group).

Supplementary Figure 2 Conditional disruption of astrocytic STAT3 inhibits PNI-induced allodynia

(a) PWT of *Stat3$^{fl//fl}$* and *Gfap-cre*;*Stat3$^{fl/fl}$* mice before (day 0) and after PNI. (n=10; $^{**}P<0.01$ versus day 0. $^{\#\#}P<0.01$ versus the ipsilateral side of *Stat3$^{fl/fl}$* mice). (b) Real-time PCR analysis of mRNA in *Stat3$^{fl//fl}$* and *Gfap-cre*;*Stat3$^{fl/fl}$* mice 14 days after PNI.

Values represent the relative ratio of mRNA (normalized to *Gapdh* mRNA) to the contralateral side of *Stat3*$^{fl/fl}$ mice (n=9, **P<0.01; ***P<0.001).

Supplementary Figure 3 Cre recombinase activity in the L4 spinal cord and DRG of *Gfap-cre;CCE* mice

(a) GFP expression in the L4 spinal cord of *Gfap-cre*;*CCE* mice (Scale bar, 200 μm). (b-f) Immunohistochemical identification of GFP-expressing cells using cell-type markers (magenta; b, GFAP; c, Sox9; d, Iba1; e, NeuN; f, APC) in the L4-SDH sections of *Gfap-cre*;*CCE* mice (Scale bar, 50 μm). (g) GFP expression in the L4 DRG of *Gfap-cre*;*CCE* mice (Scale bar, 100 μm). (h,i) Immunohistochemical identification of GFP-expressing cells using cell-type markers (magenta; h, GFAP; i, NeuN) in the L4-DRG sections of *Gfap-cre*;*CCE* mice (Scale bar, 50 μm).

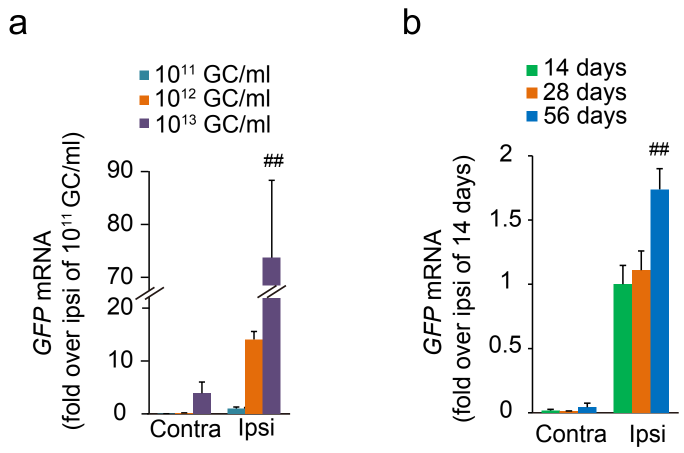

Supplementary Figure 1
Kohro et al.

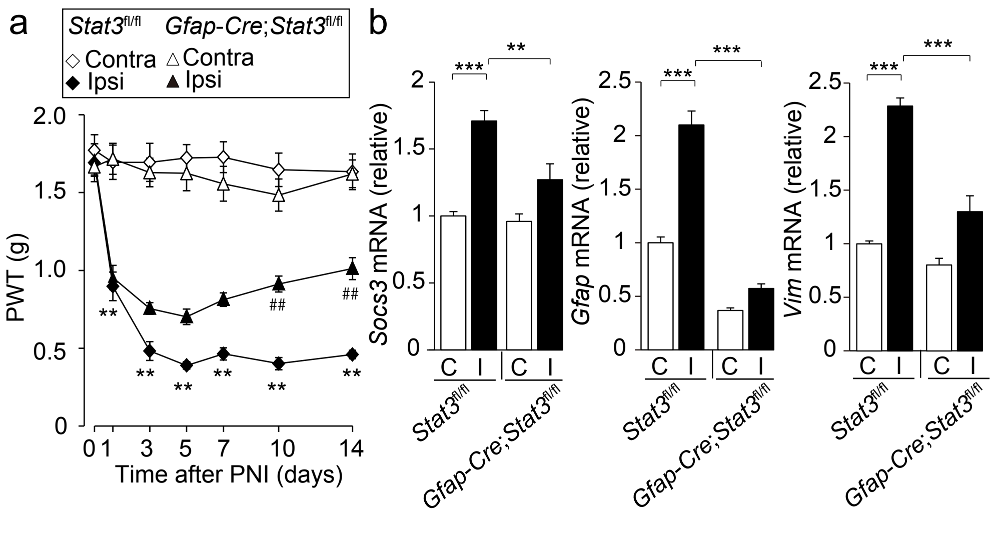

Supplementary Figure 2
Kohro et al.

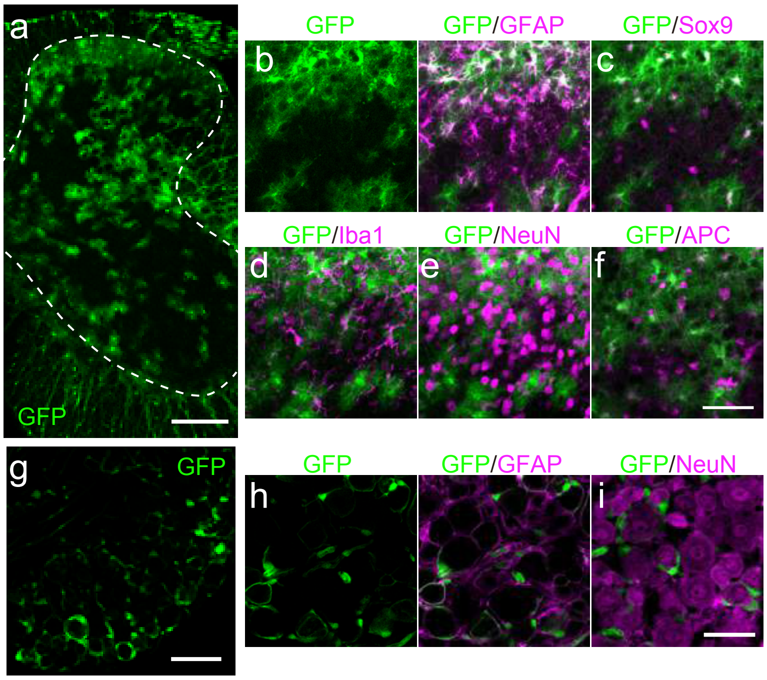

Supplementary Figure 3
Kohro et al.
